# A New Cause for Bloody Urine in Horses

**Published:** 1882-07

**Authors:** 


A New Cause for Bloody Urine in Horses.—Professor J. Lange, of Kasan, reports (*Zeitschrift f. Thiermedicin*) a very interesting case of hæmaturia in a horse, due to the presence of a parasite, probably the *filaria* of human blood. The animal was taken ill quite suddenly, refused to eat, became constipated, weak, and finally jaundiced. The pulse was feeble, temperature 39.7° C. The urine was dark-colored from blood pigment, but few red blood corpuscles being discoverable. Albumen was also present. On examining the blood from different parts of the body, under a microscope, a thread-like parasite, 1-800th inch long by 1-1850 wide, transparent and cylindrical, was seen in great numbers. A little channel seemed to run through its body. The horse, in a few days, improved, though the urine remained bloody. In about a month, it suddenly cleared up.

Professor Lange believes that the parasite is identical with that found in man, causing chyluria. He thinks also that the filaria may be a more frequent cause of hæmaturia in horses than is supposed. The filaria sanguinis hominis has been before found in the horse, and the suggestion of Professor Lange is worth the careful attention of veterinarians.

